# Diverse effects of phospholipase A2 receptor expression on LNCaP and PC-3 prostate cancer cell growth *in vitro* and *in vivo*

**DOI:** 10.18632/oncotarget.26316

**Published:** 2018-11-13

**Authors:** Markus Friedemann, Brit Nacke, Albert Hagelgans, Carsten Jandeck, Nicole Bechmann, Martin Ullrich, Birgit Belter, Christin Neuber, Olga Sukocheva, Jens Pietzsch, Mario Menschikowski

**Affiliations:** ^1^ Technische Universität Dresden, Carl Gustav Carus University Hospital Dresden, Institute of Clinical Chemistry and Laboratory Medicine, 01307 Dresden, Germany; ^2^ Helmholtz-Zentrum Dresden-Rossendorf, Institute of Radiopharmaceutical Cancer Research, Department of Radiopharmaceutical and Chemical Biology, 01328 Dresden, Germany; ^3^ School of Health Sciences, Flinders University of South Australia, Bedford Park 5042, Australia; ^4^ Technische Universität Dresden, School of Science, Faculty of Chemistry and Food Chemistry, 01062 Dresden, Germany

**Keywords:** PLA2R1 transfection, LNCaP and PC-3 prostate cancer cells, proliferation, colony formation, xenograft mouse model

## Abstract

Physiological and pathophysiological functions of the phospholipase A2 receptor 1 (PLA2R1) are still not completely understood. To elucidate PLA2R1’s function in prostate carcinoma, the receptor was ectopically overexpressed in LNCaP with silenced PLA2R1, and diminished in PC-3 cells with constitutively increased PLA2R1 expression relative to normal prostate epithelial cells. LNCaP cells were transfected to overexpress PLA2R1 (LNCaP-PLA2R1) and compared to control vector transfected cells (LNCaP-Ctrl). Alternatively, a CRISPR/Cas9-knockdown of PLA2R1 was achieved in PC-3 cells (PC-3 KD) and compared to the corresponding control-transfected cells (PC-3 Ctrl). The impact of PLA2R1 expression on proliferative and metastatic parameters was analysed *in vitro*. A pilot *in vivo* study addressed the effects of PLA2R1 in mice xenografted with transfected LNCaP and PC-3 cells. Cell viability/proliferation and motility were significantly increased in LNCaP-PLA2R1 and PC-3 Ctrl compared to LNCaP-Ctrl and PC-3 KD cells, respectively. However, levels of apoptosis, clonogenicity and cell invasion were reduced in LNCaP-PLA2R1 and PC-3 Ctrl cells. Gene expression analysis revealed an up-regulation of *fibronectin 1* (*FN1*), *TWIST homolog 1* (*TWIST1*), and *cyclin-dependent kinase 6* (*CDK6*) in LNCaP-PLA2R1. In LNCaP xenografts, PLA2R1-dependent regulation of clonogenicity appeared to outweigh the receptor’s pro-oncogenic properties, resulting in decreased tumour growth, supporting the tumour-suppressive role of PLA2R1. Alternatively, PC-3 Ctrl xenografts exhibited faster tumour growth compared to PC-3 KD cells, suggesting a pro-oncogenic effect of endogenous PLA2R1 expression. The differential growth-regulatory effects of PLA2R1 may be mediated by *FN1*, *TWIST1*, and *CDK6* expression, although further investigation is required.

## INTRODUCTION

The phospholipase A2 receptor 1 (PLA2R1) was first discovered over 20 years ago during investigation of the secreted phospholipases A2 (sPLA_2_) interaction with the corresponding candidate receptor [1, 2]. As a member of C-type lectin superfamily, PLA2R1 is a type I transmembrane receptor [3, 4]. Besides, PLA2R1 is a member of the mannose receptor family together with three other glycoproteins according to their common characteristics [5]. All representatives of this group contain a *N*-terminal cysteine-rich domain, a fibronectin type II (FNII) domain and a varying number of C-type lectin-like domains (CTLD) [3]. In contrast to other members of this family, PLA2R1 does not demonstrate any calcium-dependent lectin activity despite a tandem repeat of eight CTLDs permitting protein-protein interactions [5, 6].

PLA2R1 was recently shown to regulate apoptosis and cellular senescence in sPLA_2_-dependent and -independent manners [7–10]. Senescence and growth inhibition were induced by sPLA_2_-IIA through PLA2R1 pathway and production of reactive oxygen species (ROS) followed by a subsequent induction of DNA repair pathway in primary human fibroblasts [7]. PLA2R1 stimulated apoptosis mediating sPLA_2_-independent induction of the estrogen-related receptor α (ERRα) expression via JAK2/STAT5 signalling and mitochondrial ROS-production in MDA-MB-453 breast cancer cells [8–10]. Furthermore, PLA2R1-downregulation provoked by PLA2R1-specific shRNA treatment resulted in increased resistance to oncogenic stress–induced senescence in mammary epithelial cells *in vitro* [10].

The tumour-suppressive role of PLA2R1 was indicated in various cell types. Similar to many tumour-suppressors, PLA2R1 was down-regulated in breast and kidney cancers [11], and in melanoma cells [12]. PLA2R1 repression associated with its promoter hypermethylation was also shown in Jurkat and U937 leukemic cell lines [13], and in renal carcinoma-derived cells [11, 12]. Growth-associated colony formation in soft agar was blocked in mammary cancer cell lines MDA-MB-231 and Cama-1 constitutively expressing PLA2R1 [10]. It was described that PLA2R1-knockdown in MDA-MB-436 results in increased sizes of soft agar colonies, supporting the tumour-suppressive role of the receptor [10, 12].

However, PLA2R1 regulation of tumour growth and progression remains contradictory as PLA2R1 was found expressed at higher levels in comparison to corresponding normal cells in pancreatic and gastric cancers [12], and in leukemic blasts of patients with acute myeloid and acute lymphoid leukaemia [14]. An increased expression of PLA2R1 was also demonstrated in ovarian carcinoma effusions [15], dermatofibrosarcoma [16], and human prostate cancer cell line PC-3 [17, 18], contradicting an exclusive function of PLA2R1 as tumour-suppressor.

Therefore, the aim of the present study was to address the growth-related and cell specific role of PLA2R1 in prostate cancer cell lines LNCaP and PC-3 that differ in protein expression profiles [19, 20]. LNCaP cells with epigenetically silenced PLA2R1 expression [18] were transfected with a vector bearing the human PLA2R1 gene to up-regulate the expression. Conversely, PLA2R1-knockdown was achieved using CRISPR/Cas9 in PC-3 cells that demonstrate increased expression of PLA2R1 compared to normal prostate epithelial cells (PrEC) [18]. The impact of manipulated PLA2R1 levels on cell viability/proliferation, apoptosis, wound healing, clonogenicity, invasion, and different gene expressions was investigated. The collected data were compared with the corresponding findings in PLA2R1- and control-transfected breast cancer cell line MDA-MB-453 that was previously used to demonstrate the tumour-suppressive role of PLA2R1 [8–10]. Furthermore, *in vitro* PLA2R1 effects were compared with data obtained from a pilot study using xenograft mouse model *in vivo* with LNCaP and PC-3 cells.

## RESULTS

### Differential expression of PLA2R1 in normal and malignant prostate cells

**Figure 1:**
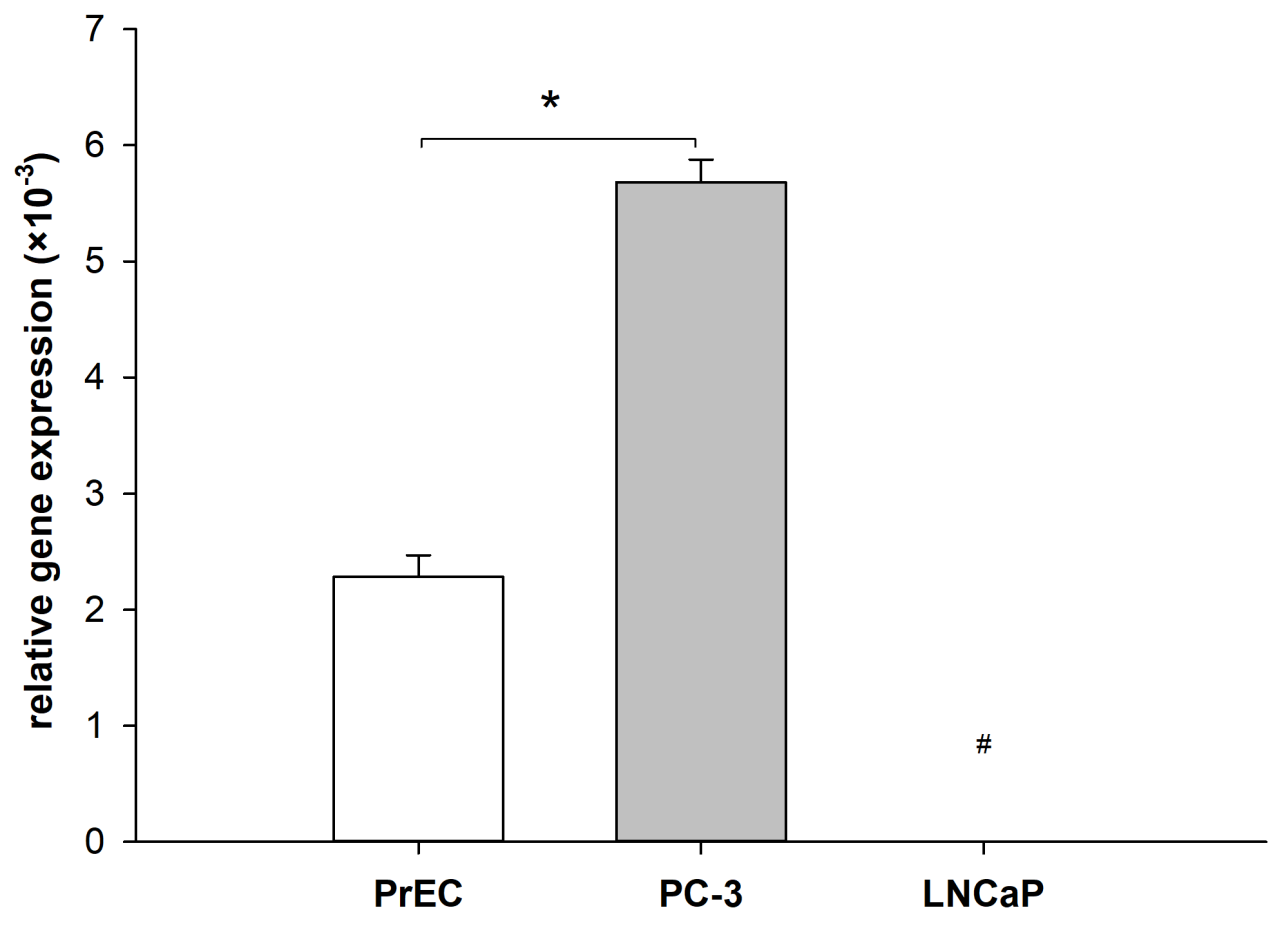
Differential expression of phospholipase A2 receptor 1 (PLA2R1) in normal and malignant prostate cells Levels of mRNA were determined using RT-qPCR. Bar graphs represent the normalized gene expression of PLA2R1 in normal prostate epithelial cells (PrEC) and prostate cancer cells (LNCaP, PC-3) with β-actin as reference gene. Results are the means ± SD of three independent experiments (biological n=3) with two technical replicates. ^#^indicates that PLA2R1 expression in LNCaP was not detected after 45 PCR cycles and therefore set to zero. ^*^ indicates significant differences with p < 0.05.

The effect of PLA2R1 expression on cancer formation and progression remains controversial as PLA2R1 was shown to have both tumour-suppressive and pro-oncogenic properties dependent on the investigated cell type [18]. To evaluate the function of PLA2R1 in prostate cells in more detail, the gene expression was analysed in normal prostate epithelial cells (PrEC) and malignant LNCaP and PC-3 prostate cancer cell lines using quantitative PCR after reverse transcription (Figure 1). Comparing to PrEC cells, the PLA2R1 mRNA level was significantly upregulated in androgen-insensitive PC-3 prostate cancer cells. We did not detect any PLA2R1 mRNA expression in androgen-sensitive LNCaP prostate cancer cells (Figure 1).

### Transfection-based overexpression of PLA2R1 in LNCaP cells and PLA2R1-knockdown in PC-3 cells

**Figure 2:**
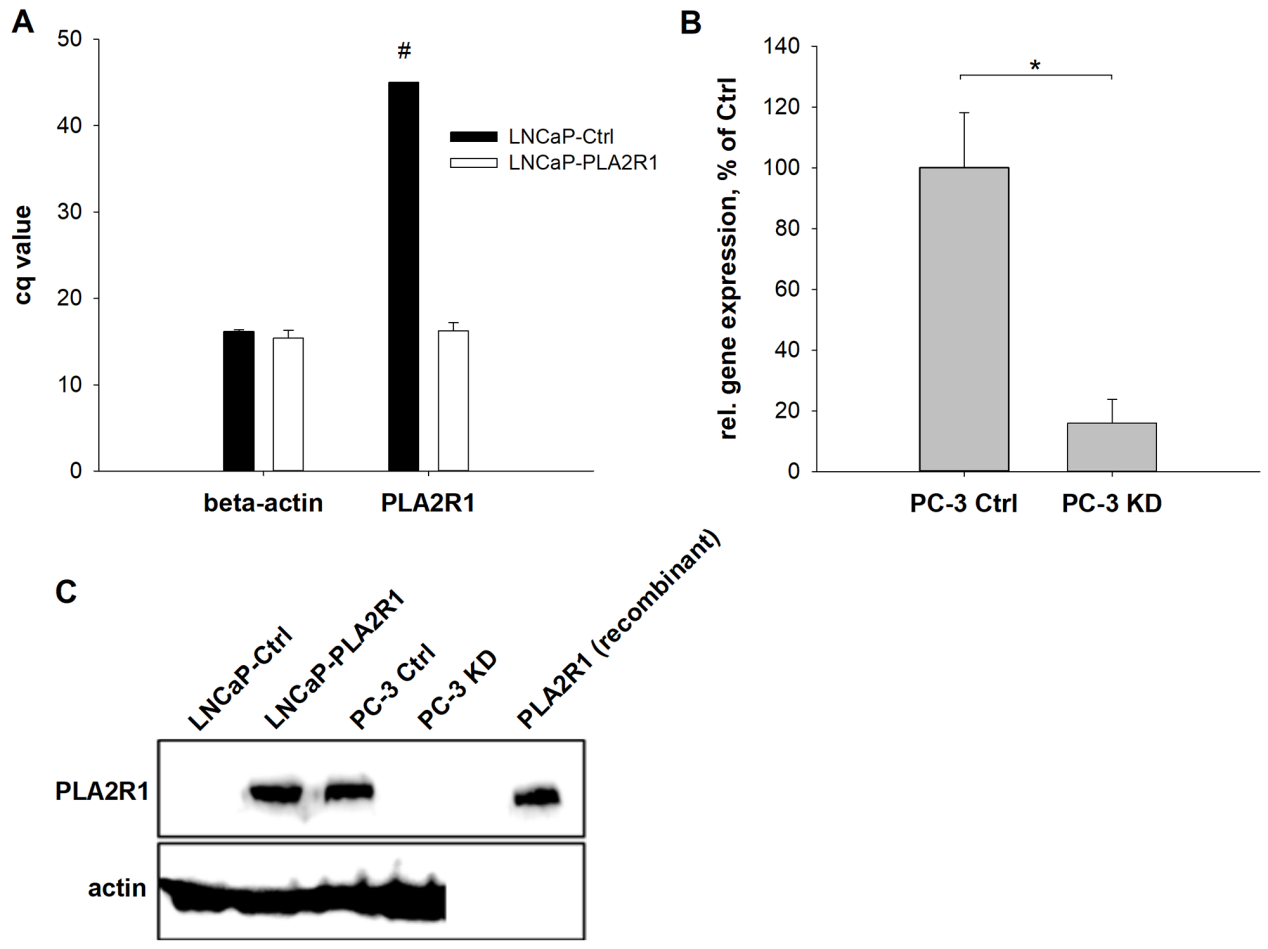
Phospholipase A2 receptor 1 (PLA2R1) expression was assessed in transfected LNCaP (LNCaP-PLA2R1) and PC-3 (PC-3 KD) cells or control vector-transfected cells (Ctrl) The amount of PLA2R1-mRNA was determined for LNCaP **(A)** and PC-3 cells **(B)** via RT-qPCR with β-actin as reference gene. Results are the means ± SD of three independent experiments (biological n=3) with two technical replicates. **(A)** Bar graphs represent the Cq values of PLA2R1 or β-actin. #indicates that PLA2R1 expression in LNCaP cells was not detected after 45 PCR cycles. **(B)** Ratio of PLA2R1 and β-actin gene expression. ^*^ indicates significant differences to the corresponding control with p < 0.05. **(C)** The protein expression of PLA2R1 was analysed by western blot with actin as reference protein and human recombinant PLA2R1 as positive control. A representative section out of three independent experiments is illustrated (biological n=3).

To establish a cell line marked by permanent PLA2R1 overexpression, LNCaP cells were transfected with a PLA2R1 plasmid vector (LNCaP-PLA2R1). Results were compared to control vector transfected LNCaP cells (LNCaP-Ctrl). Alternatively, PLA2R1 was knocked down using CRISPR/Cas9 in PC-3 cells (PC-3 KD) with endogenous levels of PLA2R1 expression (Figure 2). The expression of PLA2R1 mRNA was comparable to the level of β-actin mRNA in LNCaP-PLA2R1 (Figure 2A). Western blot data indicated the expression of PLA2R1 protein in LNCaP-PLA2R1, although neither mRNA expression nor protein synthesis of PLA2R1 was detected in LNCaP-Ctrl cells (Figure 2C). The PLA2R1 gene expression level was significantly reduced in PC-3 KD presenting only 20% of the level of control vector transfected PC-3 cells (PC-3 Ctrl; Figure 2B). Using western blot analysis, PLA2R1 protein expression was detected in PC-3 Ctrl cells, but not in PC-3 KD cells (Figure 2C).

### Differential PLA2R1 expression influences the proliferative and metastatic behaviour of prostate cancer cells

**Figure 3:**
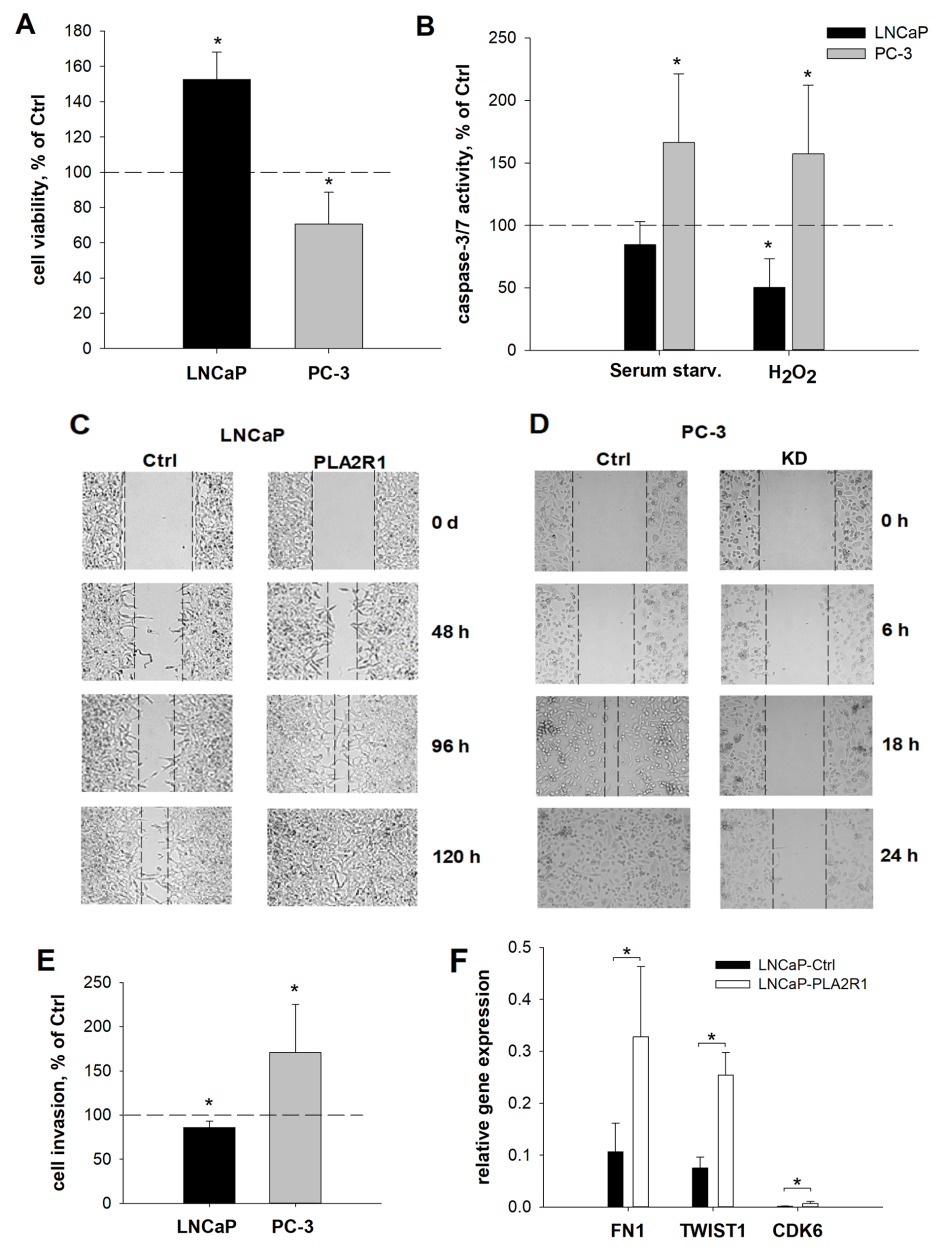
Cell viability, apoptosis, wound healing, invasion, and relative gene expression were assessed in PLA2R1-transfected LNCaP cells (LNCaP-PLA2R1) and PLA2R1-knockdown PC-3 cells (PC-3 KD) compared to control vector-transfected cells (Ctrl) Results are the means ± SD or representative illustrations of three independent experiments. **(A, B)** Results are normalized to control vector-transfected cells. **(A)** Proliferation of LNCaP was analysed using WST-1 assay after 96 h (biological n=12). **(B)** Apoptosis was stimulated by serum starvation or with hydrogen peroxide for 24 h and determined by Caspase-Glo^®^ 3/7 assay (biological n=12). **(C, D)** Culture-inserts were used to create a defined gap of 500 μm between confluent LNCaP **(C)** or PC-3 **(D)** cell layers (biological n=9). The gap width is inversely proportional to the cell motility. **(E)** Pre-starved cells were transferred to a CytoSelect™ membrane coated with collagen I and incubated for 24h. Cell invasion was determined by fluorometric quantitation (biological n=12). **(F)** The relative amount of *fibronectin 1* (*FN1*)-, *TWIST homolog 1* (*TWIST1*)- and *cyclin-dependent kinase 6* (*CDK6*)-mRNA was determined for transfected LNCaP cells with β-actin as reference gene via RT-qPCR (biological n=3). Only significant gene expression changes >2x are illustrated. ^*^ indicates significant differences to the corresponding control with p < 0.05.

**Figure 4:**
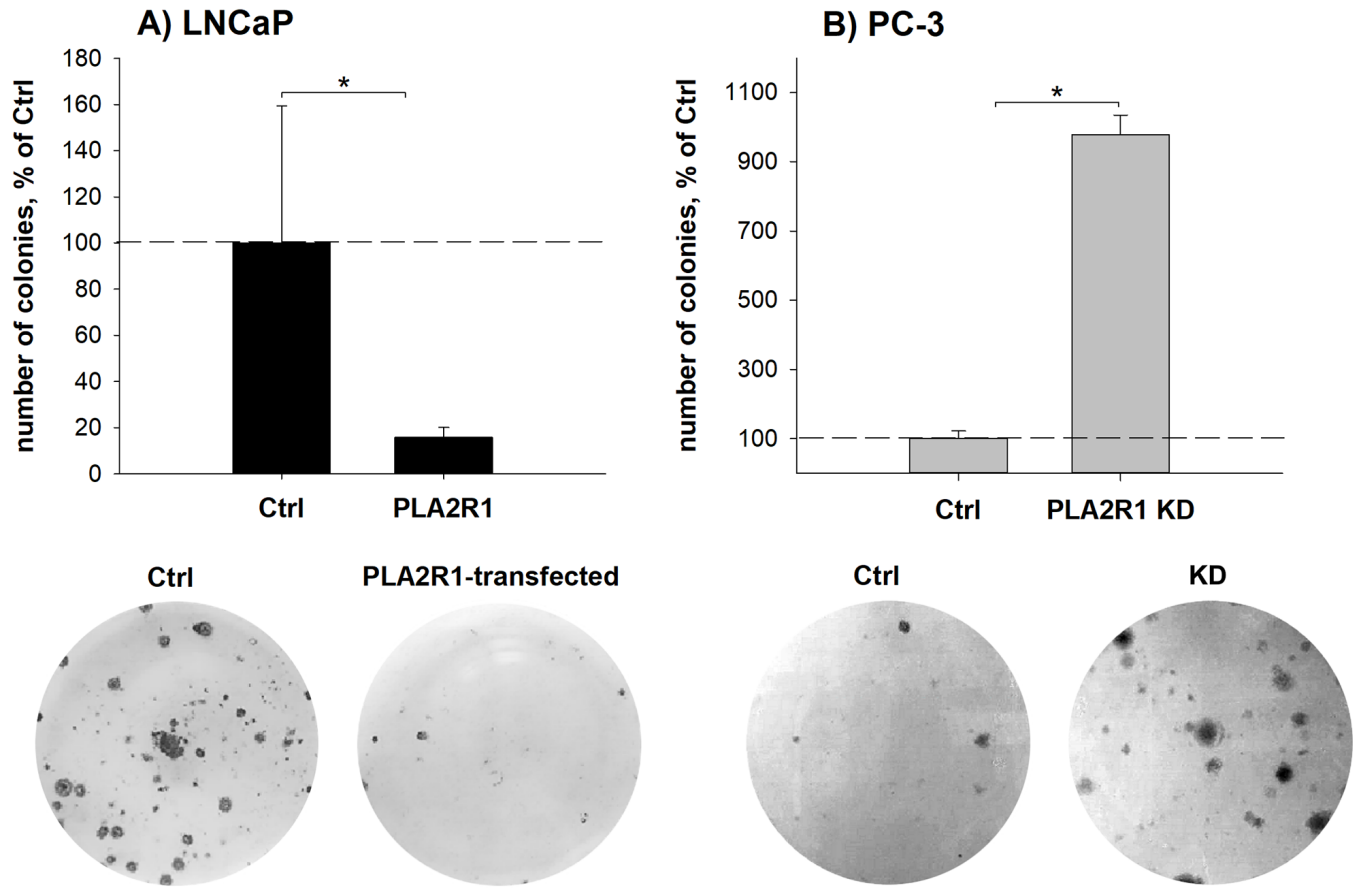
Clonogenicity was assessed in PLA2R1-transfected LNCaP cells (LNCaP-PLA2R1) and PLA2R1-knockdown PC-3 cells (PLA2R1 KD) in comparison to control vector-transfected cells (Ctrl) For clonogenic assay, LNCaP **(A)** or PC-3 cells **(B)** were incubated for 21 d, stained with crystal violet and the number of colonies (>50 cells) was determined. Representative wells and the means ± SD of three independent experiments are shown (biological n=9). ^*^ indicates significant differences with p < 0.05.

To assess the influence of different PLA2R1 expression levels, we examined cell viability/proliferation, susceptibility to cytotoxic stimuli triggered apoptosis, wound healing, cell invasion, and clonogenicity in transfected PC-3 and LNCaP cells (Figure 3 and 4). Cell viability/proliferation was significantly higher in LNCaP-PLA2R1 than in LNCaP-Ctrl cells, although the survival/growth capacity was decreased in PC-3 cells with PLA2R1-knockdown by CRISPR/Cas9 compared to the corresponding control cells (Figure 3A and Supplementary Figure 1). These data were reproduced by siRNA-knockdown of PLA2R1 in PC-3 cells (Supplementary Figure 2). Caspase 3/7 activity was significantly reduced in LNCaP-PLA2R1 cells comparing to LNCaP-Ctrl level after treatment with 100 μM of H_2_O_2_ (Figure 3B). Decreased apoptosis in LNCaP-PLA2R1 cells was confirmed by analysis of phosphatidylserine exposure on cell membranes (Supplementary Figure 3). Alternatively, caspase 3/7 activity and level of phosphatidylserine exposure were significantly increased in PC-3 KD cells compared to PC-3 Ctrl cells after serum starvation and treatment with H_2_O_2_ (Figure 3B and Supplementary Figure 3). LNCaP-PLA2R1 cells also showed faster wound healing than LNCaP-Ctrl cells. PC-3 KD cells demonstrated a decreased wound healing capability (Figure 3C/D). Collagen type I-dependent cell invasion was decreased in LNCaP-PLA2R1, and in PC-3 Ctrl cells (Figure 3E). In search for possible reasons of the differential effects of PLA2R1 expression, we analysed the gene expression profile relevant to regulation of proliferation/cell cycle-, apoptosis/senescence, and migration/invasion processes (Supplementary Table 2). *Fibronectin 1* (*FN1*) and *TWIST homolog 1* (*TWIST1*) gene expression levels were upregulated approximately three times in LNCaP-PLA2R1 compared to LNCaP-Ctrl cells (Figure 3F). *Cyclin-dependent kinase 6* (*CDK6*) gene expression was increased over four times in LNCaP-PLA2R1 compared to control cells (Figure 3F). Notably, we did not find an altered expression of these genes in PC-3 KD cells compared to control cells (data not shown). We also tested the effect of PLA2R1 on cell line capacity to form colonies. The number of observed colonies was significantly lower in LNCaP-PLA2R1 compared to LNCaP-Ctrl cells (Figure 4A). The ability to form colonies was significantly increased in PC-3 KD compared to PC-3 Ctrl cells (Figure 4B).

### Different PLA2R1 levels influence the proliferative and metastatic behaviour of MDA-MB-453 mammary cancer cells

The collected data partially contradicts previously reported information [8–10]. Thus, to verify our current transfection method applied to LNCaP cells, we also transfected the breast cancer cell line MDA-MB-453. PLA2R1 expression is silenced by promotor methylation [21–23] in MDA-MB-453 cells used as cell target in previous studies [8, 9]. PLA2R1-transfected MDA-MB-453 cells (MDA-MB-453 PLA2R1) showed stably expressed PLA2R1 mRNA (Supplementary Figure 4A) and protein levels (Supplementary Figure 4B).

We compared cell viability, apoptosis, wound healing, cell invasion and clonogenicity of MDA-MB-453 PLA2R1 and control cells (Supplementary Figure 5). The cell viability was significantly lower in MDA-MB-453 PLA2R1, along with a significantly increased susceptibility of MDA-MB-453 PLA2R1 cells to apoptosis inducing stimuli compared to MDA-MB-453 Ctrl cells (Supplementary Figure 5A and 5B). The wound healing and invasive characteristics were significantly downregulated in MDA-MB-453 PLA2R1 cells (Supplementary Figure 5C and 5D). The capacity to form colonies was also decreased by approximately 70% in MDA-MB-453 PLA2R1 compared to control cells (Supplementary Figure 5E and 5F).

### Effects of differential PLA2R1 expression on tumour xenograft growth *in vivo*

**Figure 5:**
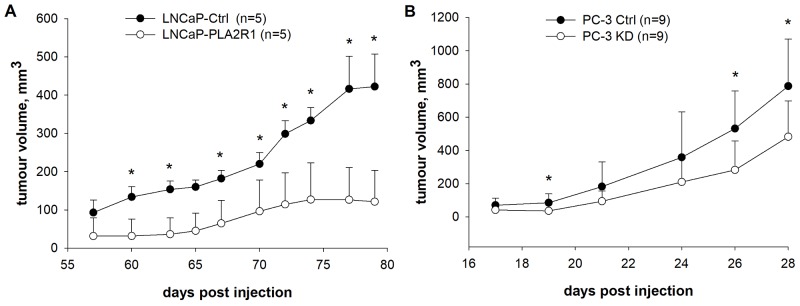
Tumour growth was tested *in vivo* in xenograft mouse models PLA2R1-transfected LNCaP (LNCaP-PLA2R1), PLA2R1-knockdown PC-3 (PC-3 KD), and control vector-transfected (Ctrl) xenografted cancer cell growth was tested and compared. **(A)** Male SCID/beige mice were subcutaneously injected with LNCaP-PLA2R1 or LNCaP-Ctrl cells in matrigel into the right hind leg (biological n=5). **(B)** Male NMRI nude mice were subcutaneously injected with PC-3 KD or PC-3 Ctrl cells in PBS into the right hind leg (biological n=9). ^*^ indicates significant differences with p < 0.05.

To confirm the observed PLA2R1 effects *in vivo*, mice were xenografted with transfected LNCaP (Figure 5A) and PC-3 cells (Figure 5B). Injection of cells in matrigel was an essential prerequisite to obtain reasonable tumour growth of LNCaP cells as injection of the cells suspended in PBS did not result in tumour formation (data not shown). Western blot analysis of tumour xenografts confirmed PLA2R1 synthesis of LNCaP-PLA2R1 and PC-3 Ctrl cells, while no PLA2R1 expression was detectable for LNCaP-Ctrl and PC-3 KD tumour specimens (Supplementary Figure 6). LNCaP-PLA2R1 cells demonstrated significantly decreased tumour growth after 60 days post injection compared to LNCaP-Ctrl cells. PC-3 originating tumour exhibited substantially faster tumour growth compared to LNCaP model. However, comparing PC-3 KD with PC-3 Ctrl cells, tumour growth of the former tended to decrease from day 19-reaching significant differences at day 26-post injection and subsequent observations (Figure 5).

## DISCUSSION

The detailed role of PLA2R1 in tumorigenesis remains unclear considering heterogeneity of the observed data in different cell lines of the same cancer type [11–16, 18]. Malignant prostate cell lines including LNCaP and PC-3 were reported to express increased levels of PLA2R1 in comparison to cell cultures derived from normal prostate tissue (PCS-440-010) [17]. However, the current study and our recently discussed data [18] indicated that PLA2R1 expression was silenced in the LNCaP cell line, but up-regulated in PC-3 cells compared to normal prostate epithelial cells (Figure 1). High degree of PLA2R1 promotor methylation was detected in LNCaP, while only minor degree of the methylation was observed in PC-3 cells. PLA2R1 was re-expressed after exposure to 5-Aza-2′-deoxycytidine (also known as Decitabine) suggesting the involvement of epigenetic mechanism in the regulation of PLA2R1 signalling in LNCaP cells [18]. We attempted to exclude methodology-associated errors (genotypic artefacts or cross contaminations) that might be involved as potential confounding factors in diversification of PLA2R1 expression in the analysed prostate cancer cell lines by short tandem repeat analysis. Notably, our results are consistent with previously reported cell line specific RNA sequencing results [22, 23]. Aiming to clarify the function of PLA2R1 in prostate cancer, we established two *in vitro* prostate cancer cell line models that include LNCaP cells with ectopically overexpressed PLA2R1 (LNCaP-PLA2R1) and PC3 cells with PLA2R1-knockdown (PC-3 KD), where wildtype PLA2R1 expression is increased relative to normal prostate epithelial cells (Figure 1).

According to our *in vitro* data, the ectopic overexpression of PLA2R1 in LNCaP supported a phenotype marked by increased cell viability/proliferation and tolerance to apoptotic stimuli (Figure 3A, 3B). The data are consistent with the improved wound healing capabilities of LNCaP-PLA2R1 cells. The wound healing assay reflects the interplay of proliferation and motility of the investigated cells and confirms a pro-oncogenic role of PLA2R1 in LNCaP-PLA2R1 cells. Upregulated *fibronectin 1* (*FN1*) can be suggested as a possible mediator of PLA2R1 pro-oncogenic signalling in LNCaP-PLA2R1 (Figure 3F). FN1 inhibition results in a significantly decreased cell adhesion of LNCaP wild-type cells [24]. FN1 overexpression stimulates cell growth and reduces apoptosis after treatment with standard chemotherapeutics in lung carcinoma [25, 26]. Accordingly, we found an upregulation of *TWIST1* (*Twist homolog 1*) in LNCaP-PLA2R1 cells, that is potentially associated with suppression of apoptosis pathways and increased cell migration [27]. LNCaP cells are androgen sensitive cells and express androgen receptors (ARs) essential for androgen-dependent tumour growth [28, 29]. The ARs signalling pathway includes expression of cyclin D and related genes responsible for cell cycle control. Interestingly, it is claimed that FN1 stimulates the expression of gonadal steroids interacting with vertebrate ARs [30]. We found an increased expression of *cyclin dependent kinase 6* (*CDK6*) in LNCaP-PLA2R1 cells. CDK6 binds to cyclin D1 for cell cycle control, but it can also bind to ARs and stimulate its transcriptional activity in the presence of dihydrotestosterone [31]. Consequently, potential tumorigenic properties of LNCaP-PLA2R1 cells might be linked to the increased expression of *FN1*, *TWIST1* and *CDK6*. Although, further studies are warranted to ensure synthesis of these proteins and confirm their possible involvement in PLA2R1 pro-oncogenic signalling in LNCaP cells.

Contrary to LNCaP, in wild-type PC-3 cells PLA2R1 is constitutively expressed and the level of PLA2R1 mRNA is higher compared to normal prostate epithelial cells (PrEC, Figure 1). Considering high selective pressure and mutation rate during tumour development, and potential metabolic burden caused by PLA2R1-overexpression, it seems unreasonable to assume that PLA2R1 has a tumour-suppressive function in PC-3. Accordingly, PLA2R1-knockdown decreased cell viability/proliferation and wound healing, but increased susceptibility to apoptotic stimuli (Figure 3A, B, D) highlighting possible pro-oncogenic PLA2R1 effects in prostate cancer cells *in vitro*. However, no significant changes in expression of *FN1, TWIST1*, and *CDK6* were detected in transfected PC-3 (data not shown). Notably, wild-type expression level of these genes is already upregulated in PC-3 (especially for *CDK6*, ∼30x) compared to LNCaP as shown by RNA sequencing data [22, 23]. Thus, the effect of forced PLA2R1 expression might be counterbalanced. Arguing our data, Quach *et al.* described an increased PC-3 cell proliferation after shRNA-induced PLA2R1-knockdown [17]. Different methods applied for PLA2R1-knockdown (shRNA vs. CRISPR/Cas9) and associated culture conditions could serve as likely explanations for the observed contradiction. Moreover, PC-3 cell line is comprised of different cell variants exhibiting strong genotypic and phenotypic heterogeneity [32]. The resulting cell responses could vary according to the applied *in vitro* culture conditions affecting differently the unknown ratio of various PC-3 cell variants [32]. To exclude the potential confounding effect of genotypic artefacts or cross contamination, short tandem repeat analysis was conducted for the analysed PC-3 cell line.

To decipher the opposite effects of PLA2R1 in LNCaP and PC-3 cells together with those described in the breast cancer cell line MDA-MB-453, we validated our transfection method and experimental procedures by transfecting MDA-MB-453 cells to overexpress PLA2R1. Endogenous PLA2R1 is silenced by hypermethylation in MDA-MB-453 cells [21]. Re-expression of the receptor is extensively discussed in literature and shown to have a tumour-suppressive function [8, 9]. In this study, the tumour-suppressive influence of PLA2R1 was confirmed. We detected decreased cell viability, lower wound healing and colony formation capabilities, but increased susceptibility to apoptotic stimuli in MDA-MB-453 cells overexpressing PLA2R1 (MDA-MB-453 PLA2R1, Supplementary Figure 5). PLA2R1 associated activation of JAK2, estrogen related receptor α (ERRα) signalling, and mitochondrial apoptosis pathways were presented as mediators of the PLA2R1 tumour-suppressive function in MDA-MB-453 cells [9]. Additionally, the mitochondrial content was elevated in PLA2R1-overexpressing MDA-MB-453 cells [9]. The baseline mitochondrial yields are significantly increased in wild-type prostate cancer cell lines LNCaP and PC-3 in comparison to PrEC [33]. The maximum mitochondrial yield was observed in LNCaP [33] given that PLA2R1 expression is silenced due to promotor hypermethylation in these cells [18]. The LNCaP mitochondrial content is higher than that of PC-3 cells, although the PC-3 PLA2R1 promotor region is methylated to a minor degree resulting in an increased expression of PLA2R1 relative to PrEC cells [18]. Thus, mitochondrial apoptosis pathway cannot serve as sufficient sole mediator of PLA2R1 biological impact.

The observed PLA2R1 inhibitory effect in LNCaP and PC-3 cells on clonogenicity is consistent with data obtained in MDA-MB-231 breast cancer cells [10]. The clonogenic assay defines the cell fraction that undergoes unlimited cell divisions retaining the ability to produce colonies [34]. Thus, proliferation and clonogenicity do not necessarily follow the same trend, describing potentially different cell fractions (stem-cell like versus non-stem like cancer cells) and aspects of cellular behaviour during tumour progression. Interestingly, stem-cell like CD44^+^/CD24^-^ cell subpopulation is relatively rare in LNCaP [35], while it is more represented in PC-3 [36] and MDA-MB-231 cells [37]. Notably, CD44^+^/CD24^-^ cell subpopulation was not detected in MDA-MB-453 [37] and BT-20 cells [38], where PLA2R1 has a consistently negative impact on both proliferation and clonogenicity [8]. However, PLA2R1 clonogenic potential and associated impact of the stem-cell like CD44^+^/CD24^-^ cell population require further detailed investigations.

To confirm our *in vitro* data, the transfected LNCaP and PC-3 cells were xenografted *in vivo*. LNCaP-PLA2R1 tumours grew slower supporting putative tumour-suppressive properties of PLA2R1. The inhibitory role of PLA2R1 in LNCaP xenografted cells is in agreement with the data observed by others with human renal cell carcinoma in *in vivo* mouse model [11]. However, PC-3 Ctrl cells displayed faster tumour growth compared to PC-3 KD *in vivo* (Figure 5). The contradictory results observed *in vitro* and *in vivo* could be associated with the different effect of the microenvironment in monolayer tumour cell culture and tumour xenografts.

We hypothesize that detected *in vitro* growth-stimulating potential of LNCaP-PLA2R1 cells might be diminished *in vivo* because of decreased clonogenicity of LNCaP-PLA2R1 cells. According to the cancer stem cell (CSC) concept, tumour growth is supported by a limited CSC population that is often presented by only about 1% of the whole tumour cell population [39]. The CSC role in regulation of PLA2R1 expression is unclear, however, it might be reflected by a decreased clonogenicity of LNCaP-PLA2R1 cells *in vitro* and weakened tumour growth *in vivo*. Non-CSCs are overrepresented in solid tumours, although they possess only a minor governance over long-term tumour survival and spreading [39]. Although less important for tumour initiation and long-term development, the non-CSC population is most likely responsible for the pro-oncogenic characteristics of LNCaP-PLA2R1 *in vitro* cell viability/proliferation, apoptosis, and wound healing. Considering serious differences between CSC and non-CSC general cancer cell populations, the clonogenicity as a long-term assayed (21-day growth) *in vitro* characteristic corresponds better with *in vivo* data. Presumably, PLA2R1-linked clonogenicity (stemness) of LNCaP subpopulations is a crucial factor required for tumour development *in vivo*. Interestingly, the fraction of the CSC-like CD44^+^/CD24^-^subpopulation differs among various prostate cancer cell lines and comprises only 0.04% of LNCaP cells [35] compared to ∼11% of PC-3 cells [36]. Thus, the inhibiting impact of PLA2R1 expression on the CSC subpopulation could be more pronounced in LNCaP than in PC-3 cells. The increased tumour growth of PC-3 cells with endogenous PLA2R1 expression *in vivo* could therefore indicate PLA2R1 effects in CD44^+^/CD24^-^ subpopulation that require further confirmation.

Furthermore, the mouse xenograft model potentially provides superior information about tumour formation *in vivo* that considers cell-matrix interactions, the influence of initial cell adhesion and subsequent tumour growth, which cannot be fully replicated in *in vitro* clonogenic assays using plastic surfaces. PLA2R1 can interact with collagen type I and other extracellular matrix components via its CLTD1-2 and FNII domains and its indirect interaction with β1 integrin via collagen I can influence cell proliferation [40, 41]. However, the outcome of this interaction can be influenced by the corresponding α integrin subunit resulting in opposite effects on proliferation and differentiation [42]. For instance, integrin α1β1 is discussed to promote cell proliferation, whereas α2β1 can inhibit cell growth [22, 23, 42–44]. RNA sequencing data shows no or severely limited gene expression of ITGA1 in wild-type LNCaP cells, whereas ITGA1-gene expression is found in PC-3 cells [22, 23]. By contrast, ITGA2 is expressed in both prostate cancer cell lines [22, 23]. Suggestively, the functional interaction between PLA2R1 and β1 integrin may favour ITGA2 in LNCaP cells due to the lack of ITGA1-expression resulting in a decreased tumour growth and tumour-suppressive role of PLA2R1 *in vivo*. Alternatively, representing a possible explanation of PLA2R1’s pro-oncogenic effect *in vivo*, the indirect interaction between PLA2R1 and α1β1 via collagen type I may occur in PC-3 cells expressing ITGA1. Further immunohistochemical analysis of tumour xenografts has to be conducted to investigate the interactions of PLA2R1 *in vivo*.

## MATERIALS AND METHODS

### Cell culture and incubation

Human prostate epithelial cells (PrEC; Cambrex Bio Science, Walkersville, MD, USA) and prostate cancer cell lines (PC-3 and LNCaP; DSMZ, German Collection of Microorganisms and Cell Cultures, Braunschweig, Germany) were cultured as previously described [45]. The human mammary cancer cell line MDA-MB-453 (DSMZ) was cultured in DMEM (4.5 g/l glucose, w/o *L*-glutamine and sodium pyruvate; Lonza, Cologne, Germany) supplemented with 20% FCS at 37°C in a humidified atmosphere of 5% CO_2_. Transfected LNCaP and MDA-MB-453 cells were cultured in the medium supplemented with 500 mg/ml G418 Sulfate (Thermo Fisher Scientific, Waltham, MA, USA). Transfected PC-3 cells were grown in the medium supplemented with 2.5 mg/ml puromycin dihydrochloride (Santa Cruz Biotechnology, Inc., Dallas, Texas, USA). The culture medium was changed every 2 - 3 days. Cells were passaged before reaching confluence using Trypsin/EDTA solution (PromoCell, Heidelberg, Germany). Cell lines were validated using the ATCC cell line authentication service (LGC-ATCC, Middlesex, UK) and were frequently tested for mycoplasma contamination (MycoAlert™ Mycoplasma Detection Kit, Lonza).

### Transfection

LNCaP and MDA-MB-453 cells were transfected using the ViaFectTM Transfection Reagent (Promega, Madison, WI, USA) and an optimized expression vector for phospholipase A2 receptor 1 (NP_031392.3; GenScript, Piscataway, NJ, USA), or a control vector (GenScript). The transfected cells were selected using 500 mg/mL G418 Sulfate. Survived cells were used in subsequent experiments.

For PLA2R1-knockdown with CRISPR/Cas9, the double nickase strategy with substantially reduced off-target activity was utilized to specifically decrease PLA2R1 expression [46]. PC-3 cells were transfected with PLA2R- (sc-411636-NIC, Santa Cruz) or Control Double Nickase Plasmids (sc-437281, Santa Cruz) as described in the protocol provided by the manufacturer using 2 μg of plasmid DNA with a ratio of 6:1 for ViaFectTM Transfection Reagent and plasmid DNA correspondingly. Transfected PC-3 cells were selected using 2.5 mg/ml puromycin dihydrochloride (Santa Cruz). Clonal selection was performed by diluting the surviving cells in a 96 well plate. Transfected single cell clones were verified by GFP fluorescence and used separately in subsequent experiments. The minimum effective G418 Sulfate or puromycin dihydrochloride concentration was defined in separate experiments assessing kill curve titration of the agents and wild-type LNCaP, MDA-MB-453, and PC-3 cells.

To achieve siRNA-knockdown of PLA2R1 in PC-3 cells, three different siRNA duplexes containing PLA2R1 Trilencer-27 Human siRNA (# SR307882, OriGene Technologies, Inc., Rockville, MD, USA) were mixed in equal parts. The siRNA mix was diluted in Opti-MEM^®^/Reduced Serum Medium (Thermo Fisher Scientific), added to siTran 1.0 Transfection Reagent (OriGene Technologies, Inc.), and applied in a final concentration of 3.3 nM each as recommended by the manufacturer. After 4 h incubation of the cells within a humidified atmosphere (37°C, 5% CO_2_), 1.25 volume parts of RPMI with 20% FCS were added. Then cells were further incubated for 48 h.

### RNA isolation and reverse transcription quantitative polymerase chain reaction (RT-qPCR)

Total RNA was isolated using QIAzol Lysis Reagent (QIAGEN GmbH, Düsseldorf, Germany) and miRNeasy Mini Kit (QIAGEN) with on-column DNase digestion (RNase-Free DNase Set, QIAGEN) as described in the manufacturer’s protocol.

Reverse transcription was performed for 45 min at 42°C and 5 min at 99°C utilizing 2.5 U MuLV Reverse Transcriptase (Thermo Fisher Scientific), 1 U Protector RNase inhibitor (Sigma-Aldrich), 0.125 μM Oligo(dT)_15_ primer (Promega), 1 mM MgCl_2_ solution (Sigma-Aldrich), 0.4 mM dNTP mix (Promega), 1×PCR Buffer without MgCl_2_ (Sigma-Aldrich), and 100 ng RNA template with a final reaction volume of 20 μL.

Quantitative PCR was conducted using the Rotor-Gene Q cycler and Rotor-Gene SYBR Green PCR Kit (Qiagen) according to the manufacturer’s protocol with β-actin as reference gene. Applied primer pairs (Supplementary Table 1) were used in a final concentration of 0.4 μM. Cycling conditions involved 5 min at 95°C followed by 45 cycles of 5 s at 95°C, and 10 s at 59°C.

RNA isolation and RT-qPCR were carried out using three separate cell culture passages, each measured twice (biological n=3, technical n=2).

### Protein isolation, SDS-PAGE and Western Blot

Preparation and Western Blot analysis of tissue extracts followed protocols published elsewhere [46, 47]. To isolate proteins from cell culture experiments, cells were lysed with CellLytic M lysis buffer (Sigma-Aldrich) supplemented with Protease Inhibitor Cocktail (Sigma Aldrich, P8340). The protein concentrations were determined by bicinchoninic acid assay (Sigma-Aldrich).

During Western Blot analysis, 40 μg protein per lane was supplemented with ClearPAGE LDS Sample Buffer (C.B.S. Scientific, Del Mar, CA, USA) and heated for 5 min at 100°C at reducing conditions (5% 2-mercaptoethanol, Sigma Aldrich). PLA2R1 human recombinant protein (TP313576, OriGene) was used as positive control. Proteins were separated with ClearPAGE 10 % sodium dodecylsulfate polyacrylamide gels (C.B.S. Scientific). The samples were transferred to PVDF membranes (Fisher Scientific). The membranes were blocked for 1 h with 5% skimmed milk powder, 2% BSA, and 0.1% Tween^®^ 20 (Serva, Heidelberg, Germany) in TBS. Recombinant rabbit monoclonal antibody (AB; 180 kDa; ab211573, Abcam, Cambridge, UK) was used as primary AB (1:500) and a horseradish peroxidase conjugated goat anti-rabbit IgG (sc-2004, Santa Cruz) as secondary AB (1:5000) for immunochemical detection of PLA2R1. Actin was detected using a 1:1000 dilution of primary mouse monoclonal AB (42 kDa; MAB1501R, Merck Millipore, Darmstadt, Germany) and a 1:5000 dilution of secondary horseradish peroxidase conjugated goat anti-mouse IgG (sc-2005, Santa Cruz). The membranes and primary AB were incubated overnight at 4°C. The secondary AB was incubated for 1 h at room temperature. Membranes were washed three times in TBST between the incubation steps. The blot was incubated with SuperSignal™ West Femto/Pico Chemiluminescent Substrate (Thermo Fisher Scientific) and luminescence was detected using the G:BOX Chemi XL1.4 system (Syngene, Cambridge, UK). Protein isolation, SDS-PAGE and Western Blot were carried out three times using separate cell culture passages (biological n=3).

### WST-1 cell viability assay

To analyse cell viability, 100 μL per well of 5×10^3^ LNCaP, MDA-MB-453 or PC-3 cells was seeded to a 96-well microtiter plate. After four-day incubation, 10 μL of the cell proliferation reagent of WST-1 Cell Proliferation Kit II (Sigma-Aldrich) was added to each well and incubated for further 2 h at 37°C. The absorption was measured at 450 nm. The WST-1 assay was carried out as quadruplicate and repeated by two additional independent experiments using separate cell culture passages resulting in biological n=12.

### Apoptosis assays

The caspase-3/7 activities were determined using Caspase-Glo^®^ 3/7 assay (Promega). 1.5×10^4^ LNCaP, MDA-MB-453 or PC-3 cells were seeded in 100 μL complete medium in a 96-well microtiter plate and incubated for 24 h within a humidified atmosphere (37°C, 5% CO_2_). The medium was removed, replaced by 100 μL starving medium or 100 μM hydrogen peroxide in 100 μL starving medium to induce apoptosis and incubated for 24 h. The caspase-3/7 activity was estimated according to the manufacturer’s protocol. Luminescence of each sample was measured using Wallac Victor 3 Multi-Label Microplate Reader (PerkinElmer, Waltham, MA, USA). Caspase-Glo^®^ 3/7 assay was carried out as quadruplicate and repeated by two additional independent experiments using separate cell culture passages resulting in biological n=12.

Time-dependent translocation of phosphatidylserine to the outer cell membrane and membrane integrity were analysed by RealTime-Glo™ Annexin V Apoptosis and Necrosis Assay (Promega) in adherent cells. 1×10^4^ LNCaP or PC-3 cells diluted in 100 μL complete medium were seeded into 96-well microtiter plate and incubated for 24 h within a humidified atmosphere (37°C, 5% CO_2_). The detection reagent was prepared according to the manufacturer’s protocol. The medium was removed and replaced by 100 μL of 200 μM hydrogen peroxide (H_2_O_2_) and 100 μL detection reagent in CO_2_-independent medium. Luminescence (apoptosis) and fluorescence (necrosis; Ex: 485 nm/Em: 535 nm) were measured every hour during 48 h using Wallac Victor 3 Multi-Label Microplate Reader (PerkinElmer) at 37 °C. Every assay sample was tested in triplicates and repeated in two additional independent experiments using separate cell culture passages (n=9).

### Wound healing assay

To estimate cell motility, 70 μL of 7×10^4^ cells in medium were transferred to each well of a 2-Well Culture-Insert (Ibidi GmbH, Planegg/Martinsried, Germany) and incubated until confluence was reached. The insert was removed and the wound healing/cell motility within the 500 μm cell free gap was registered by taking a picture every 24 h (MDA-MB-453 and LNCaP) or every 8 h (PC-3) on a phase contrast microscope (Nikon TMS-F, Tokyo, Japan; 10x objective) until the gap was completely closed. The gap width is inversely proportional to the cell motility as partial aspect of the migration capacity. Wound healing assay was carried out as triplicate and repeated by two additional independent experiments using separate cell culture passages resulting in biological n=9.

### Cell invasion assay

To analyse cell invasion, the CytoSelect™ 96-Well Cell Invasion Assay, Collagen I coating, and fluorometric quantitation (Cell Biolabs, Inc., San Diego, CA, USA) was utilized according to the manufacturer’s description with 1×10^5^ pre-starved cells per well. Cell invasion assay was carried out as quadruplicate and repeated by two additional independent experiments using separate cell culture passages resulting in biological n=12.

### Clonogenic assay

To assess clonogenicity, 500 cells per 3 ml medium were incubated in a 6-well Corning^®^ Costar^®^ cell culture plate (Sigma-Aldrich) for 21 days. The cells were washed with PBS and fixed for 5 min with 1:1 solution of PBS and methanol, and 10 min with 100% methanol. The cells were stained with crystal violet solution (Sigma-Aldrich) for 15 min and washed three times with water. The formed cell colonies were counted using a phase contrast microscope. A cell colony was defined as a compact aggregation comprising more than 50 cells. Clonogenic assay was carried out as triplicate and repeated by two additional independent experiments using separate cell culture passages resulting in biological n=9.

### Animal experiments

All animal experiments were carried out at the Helmholtz-Zentrum Dresden-Rossendorf according to the guidelines of the German Regulations for Animal Welfare approved by the local Animal Ethics Committee for Animal Experiments (Landesdirektion Dresden; AZ 24-9168.21-4/2004-1). Mice were housed in a pathogen-free facility with *ad libitum* access to food and water.

Male SCID/beige mice (CB17.Cg-*Prkdc*^*scid*^*Lyst*^*bg-J*^/Crl, T- and B-cell-deficient, NK-cell-defective, white-haired, Charles River; aged 6 to 8 weeks) were subcutaneously injected with matrigel solution (50%, solved in PBS; 50 μL) or with PBS only (biological n = 5 each) containing 5×10^6^ PLA2R1-vector or control transfected LNCaP cells into the right hind leg. Male NMRI-nude mice (Rj:NMRI-*Foxn1*^*nu*^, T-cell-deficient, hairless, Janvier Labs, Le Genest-Saint-Isle, France¸ NMRI nu/nu; aged 6 to 8 weeks) were subcutaneously injected with 2×10^6^ PLA2R1-knockdown or control PC-3 cells (biological n = 9 each) in PBS (50 μL) into the right hind leg. Tumour size was monitored three times a week by caliper measurements and tumour volume was calculated using the formula V = π / 6 × (tumour length × tumour width^2^).

Tumour-bearing mice were sacrificed by cervical dislocation when tumours reached a volume of 400 to 700 mm^3^ for slowly growing control vector transfected LNCaP (LNCaP-Ctrl) cells; or after 112 days post-injection for PLA2R1-transfected LNCaP (LNCaP-PLA2R1) cells showing stagnating tumour volume of approximately 200 mm^3^. Mice were sacrificed when tumours reached a volume of 800 to 1300 mm^3^ with rapidly growing PC-3 cells. Individual animals in each group were sacrificed at earlier time points if necrotic tumour/skin lesions and/or bleeding were detected.

Animal experiments were planned as a pilot study (information survey). No values have been excluded, no method of randomization was used, and no blinding was done.

### Data analysis

‘Center values’ were defined as means with standard deviation as error indication. Normal distribution was analysed by Shapiro-Wilk test. Variance was estimated using *F*-test. Data were analysed by two-tailed and unpaired Student’s *t* test (normally distributed, homoscedastic) or Mann-Whitney Rank Sum test (non-normally distributed) to calculate the indicated P values. Differences were considered significant at P < 0.05.

## CONCLUSIONS

Our data indicate complex and highly cell-specific functions of PLA2R1 exhibiting both pro-oncogenic (PC-3) and tumour-suppressive characteristics (LNCaP and MDA-MB-453) in different cancer cells. We suggest that PLA2R1-regulated expression of *FN1*, *TWIST1*, and *CDK6* might be accountable for the cell type-dependent impact of PLA2R1 in tumour cell survival and growth.

## SUPPLEMENTARY MATERIALS FIGURES AND TABLES




