# Supplementary material for: Diverse effects of phospholipase A2 receptor expression on LNCaP and PC-3 prostate cancer cell growth in vitro and in vivo
Source: Oncotarget. 2018 Nov 13;9(89):35983–96. doi: 10.18632/oncotarget.26316 (PMC6267604; doi:10.18632/oncotarget.26316)
Supplement: Supplementary file 1 [file oncotarget-09-35983-s001.pdf]

Diverse effects of phospholipase A2 receptor expression on LNCaP and PC-3 prostate cancer cell growth *in vitro* and *in vivo*

SUPPLEMENTARY MATERIALS

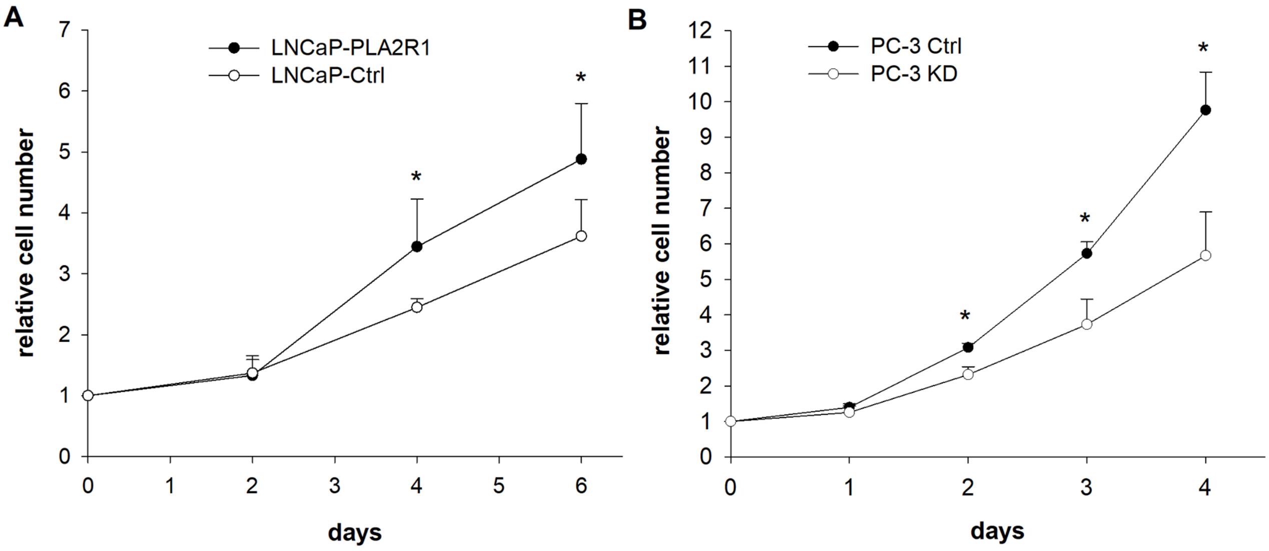

Supplementary Figure 1: Growth curve analysis of PLA2R1-transfected LNCaP cells (LNCaP-PLA2R1) and PLA2R1-knockdown PC-3 cells (PC-3 KD) compared to control vector-transfected cells (Ctrl). Results are the means ± SD of three independent experiments (biological n=3) with two technical replicates. **(A)** $2^{*}10^{5}$ LNCaP cells were seeded in 6-well plates and analysed for 6 days. **(B)** $5^{*}10^{4}$ PC-3 cells were seeded in 6-well plates and analysed for 4 days. * indicates significant differences to the corresponding control with $p < 0.05$.

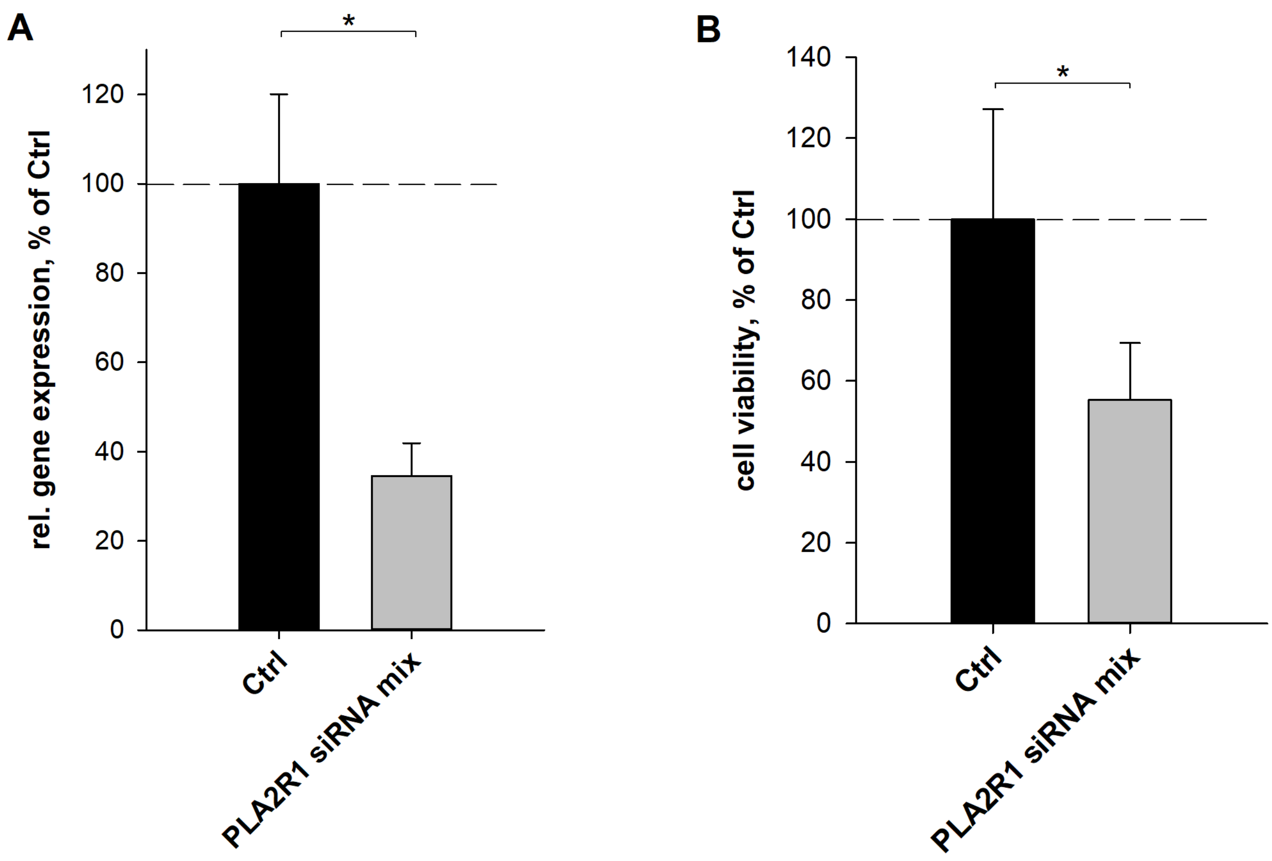

Supplementary Figure 2: Phospholipase A2 receptor 1 (PLA2R1) expression and cell viability was assessed in PC-3 cells treated with a PLA2R1 siRNA mix or scrambled siRNA (Ctrl). PC-3 cells were incubated with a PLA2R1 siRNA mix or scrambled siRNA for 48 h. Results are the means ± SD of three independent experiments (biological n=3) with three technical replicates. **(A)** Bar graphs represent the normalized gene expression of PLA2R1 analysed by RT-qPCR with GAPDH as reference gene. **(B)** Cell viability was assessed vial XTT assay. * indicates significant differences to the corresponding control with $p < 0.05$.

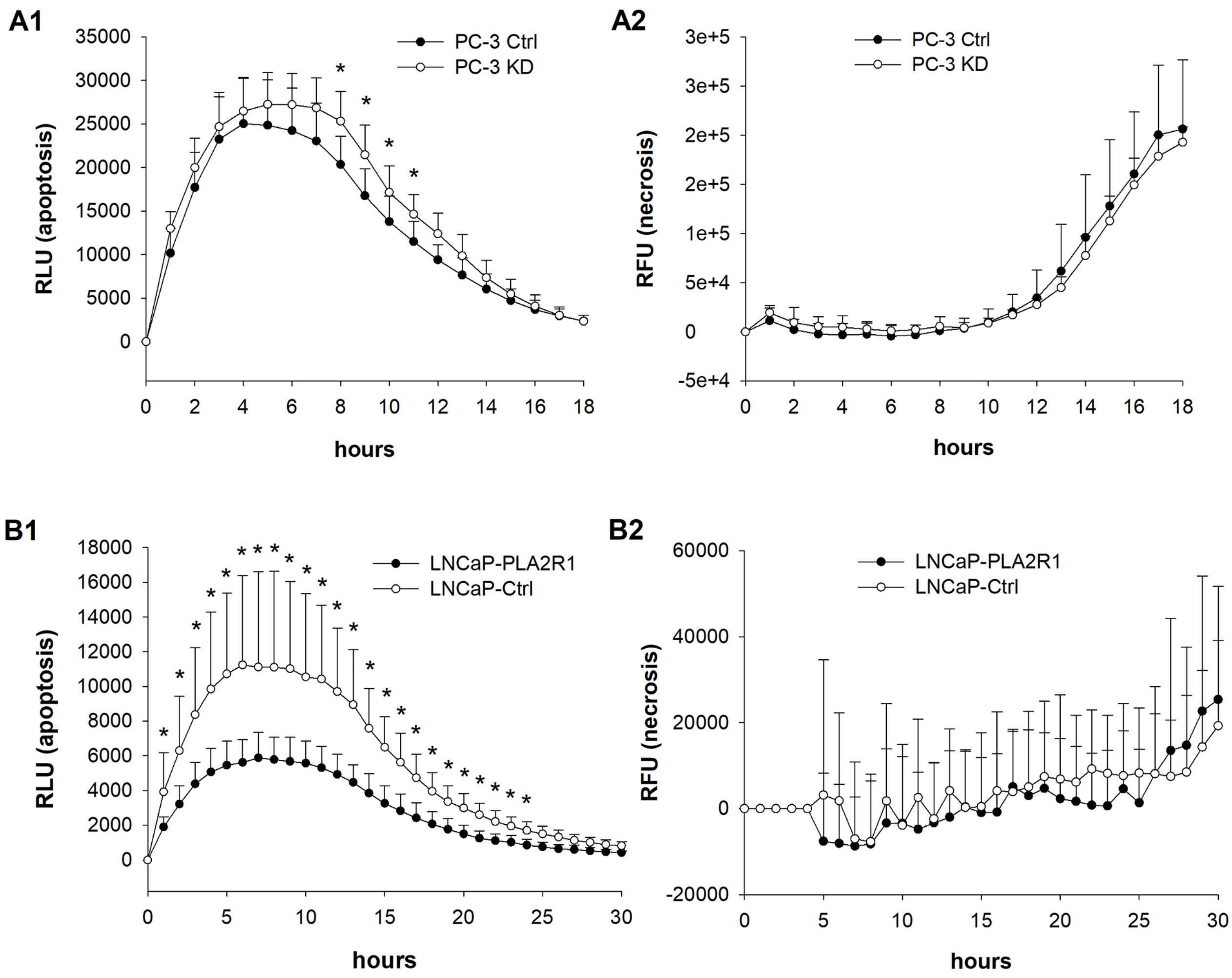

Supplementary Figure 3: Time-dependent translocation of phosphatidylserine and membrane integrity were assessed in PLA2R1-transfected LNCaP cells (LNCaP PLA2R1) and PLA2R1-knockdown PC-3 cells (PC-3 KD) compared to control vector-transfected cells (Ctrl). Results are the means ± SD of three independent experiments (biological n=9). Apoptosis was stimulated by hydrogen peroxide and determined by RealTime-Glo™ Annexin V Apoptosis and Necrosis Assay. Results are presented relative to the first measurement. Relative luminescence units (RLU) indicate exposure of phosphatidylserine and magnitude of apoptosis progression for PC-3 **(A1)** and LNCaP cells **(B1)**. Relative fluorescence units (RFU) reflect cell membrane integrity and necrosis for PC-3 **(A2)** and LNCaP cells **(B2)**. * indicates significant differences to the corresponding control with $p < 0.05$.

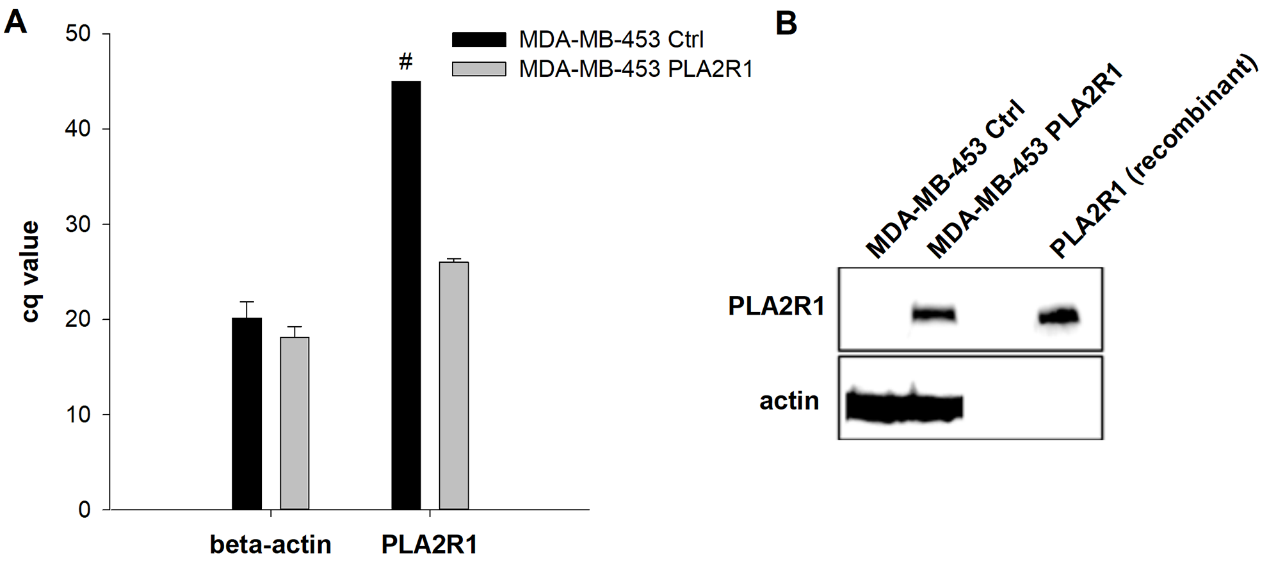

Supplementary Figure 4: PLA2R1 expression was tested in PLA2R1-transfected MDA-MB-453 (MDA-MB-453 PLA2R1) or control vector-transfected cells (MDA-MB-453 Ctrl). **(A)** The relative amount of PLAR1-mRNA was determined for MDA-MB-453 cells compared with β-actin as reference gene via RT-qPCR (mean ± SD, biological n=3, technical n=2). #indicates that PLA2R1 expression in MDA-MB-453 cells was not detected after 45 PCR cycles. **(B)** The protein expression of PLA2R1 was analysed by Western Blot with actin as reference protein and human recombinant PLA2R1 as positive control. A representative section out of three independent experiments is illustrated.

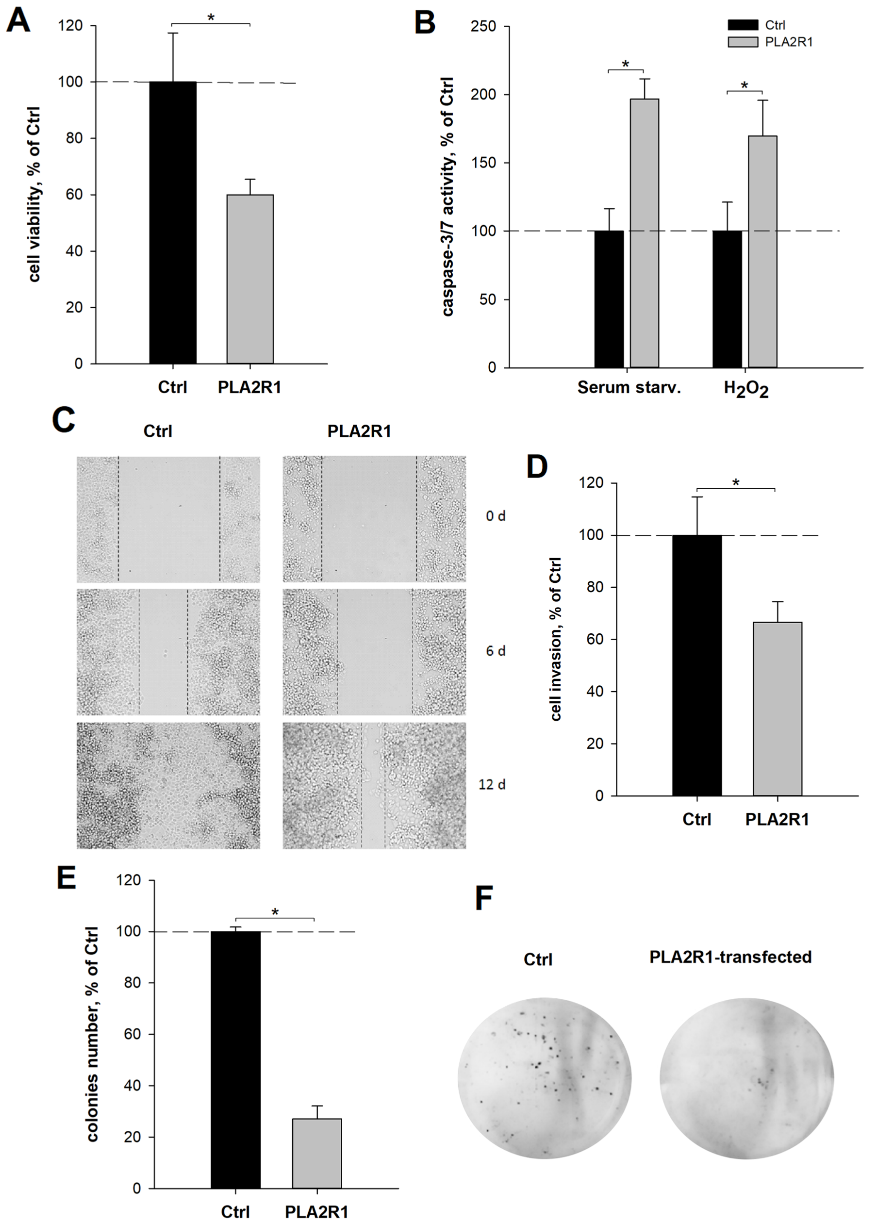

Supplementary Figure 5: Biological responses were tested in PLA2R1-transfected MDA-MB-453 cells (PLA2R1) and compared to control vector-transfected cells (Ctrl). Results are the means ± SD or representative illustrations of three independent experiments. **(A)** Cell viability was analysed by WST-1 assay (biological n=12). **(B)** Apoptosis was stimulated by serum starvation or with hydrogen peroxide and determined by Caspase-Glo® 3/7 assay (biological n=12). **(C)** Wound healing. Culture-inserts were used to create a defined gap of 500 µm between confluent cell layers (biological n=9). The gap width is inversely proportional to the cell motility. **(D)** Pre-starved cells were transferred to a CytoSelect™ membrane coated with collagen I and incubated for 24 h. Cell invasion was determined by fluorometric quantitation (biological n=12). **(E, F)** Clonogenic assay. Cells were incubated for 21 d, stained with crystal violet and the number of colonies (>50 cells) was determined (biological n=9). * indicates significant differences with $p < 0.05$.

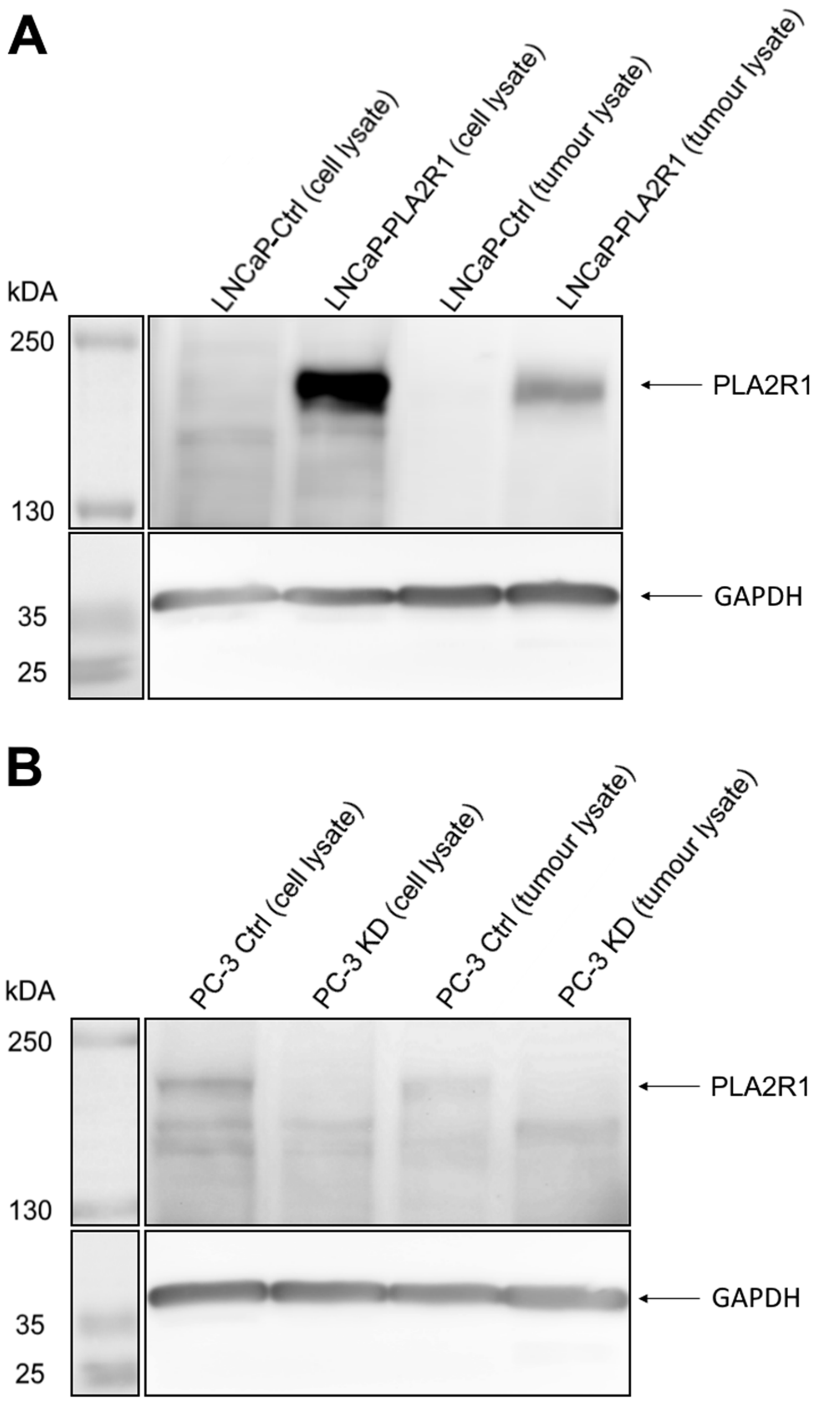

Supplementary Figure 6: Phospholipase A2 receptor 1 (PLA2R1) protein synthesis was assessed in transfected LNCaP (LNCaP-PLA2R1) and PC-3 PLA2R1-knockdown cells (PC-3 KD) or control vector-transfected cells (Ctrl) in lysates from cell culture and tumour xenografts. The protein expression of PLA2R1 was analysed for cell culture and tumour lysates of transfected LNCaP **(A)** and PC-3 cells **(B)** by western blot with GAPDH as reference protein. A representative section out of two independent experiments is illustrated (biological n=2).

Supplementary Table 1: Primer sequences of utilized primers for reverse transcription quantitative polymerase chain reaction

Gene	Forward primer	Reverse primer	Product size (bp)
PLA2R1 (vector)	GCAGCTTCACCTTTTGGAAG	CACTGCACAGCTCAGGATGT	168
PLA2R1 (genomic)	AGAGATGTGAAACCCAAGA	TCACAGTTCTTTCTCTTCC	325
PLA2R1 (CRISPR/ Cas9)	CCATCAGTGGCATCATGAATGTA	GAATGTGCCTCACTCCAAGA	219
β-actin	CACCACACCTTCTACAATGAGC	CAGAGGCGTACAGGGATAGC	178
FN1	CCATAGCTGAGAAGTGTTTTG Sigma #8022339161-10/0	CAAGTACAATCTACCATCATCC Sigma #8022339161-10/1	103
TWIST1	CTAGATGTCATTGTTTCCAGAG Sigma #8022339161-20/0	CCCTGTTTCTTTGAATTTGG Sigma #8022339161-20/1	136
CDK6	Biomol VHPS-1762	Biomol VHPS-1762	234

Supplementary Table 2: List of analysed genes from Qiagen RT^2 Profiler™ PCR Array Human Cellular Senescence.

See Supplementary File 1
